# Preparation and Characterization of Amino Acids-Based Trimethoprim Salts

**DOI:** 10.3390/pharmaceutics4010179

**Published:** 2012-02-16

**Authors:** Amr ElShaer, Peter Hanson, Tony Worthington, Peter Lambert, Afzal R. Mohammed

**Affiliations:** Aston Pharmacy School, Aston University, Aston Triangle, Birmingham B4 7ET, UK

**Keywords:** trimethoprim, salt formation, fourier transform infrared, 1H nuclear magnetic resonance, thermogrametric analysis, Pseudomonas aeruginosa, minimum inhibitory concentration studies

## Abstract

Trimethoprim (TMP) is a dihydrofolate reductase (DHFR) inhibitor which prevents the conversion of dihydrofolic acid into tetrahydrofolic acid, resulting in the depletion of the latter and leading to bacterial death. Oral bioavailability of TMP is hindered by both its low solubility and low permeability. This study aims to prepare novel salts of TMP using anionic amino acids; aspartic and glutamic acid as counter ions in order to improve solubility and dissolution. TMP salts were prepared by lyophilisation and characterized using FT-IR spectroscopy, proton nuclear magnetic resonance (^1^HNMR), Differential Scanning Calorimetry (DSC) and Thermogravimetric analysis (TGA). Both the amino acids formed salts with TMP in a 1:1 molar ratio and showed a 280 fold improvement in solubility. Investigation of the microbiological activity of the prepared salts against TMP sensitive* Escherichia coli* showed that the new salts not only retained antibacterial activity but also exhibited higher zone of inhibition which was attributed to improved physicochemical characters such as higher solubility and dissolution. The results are an important finding that could potentially impact on faster onset of antibacterial activity and reduced therapeutic dose when administered to patients. Studies are underway investigating the effect of ion-pairing TMP with amino acids on the permeability profile of the drug.

## 1. Introduction

**Figure 1:**
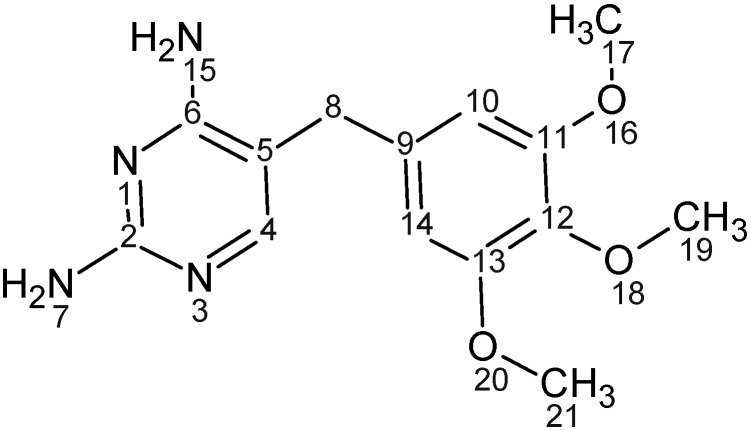
Trimethoprim chemical structure.

Trimethoprim (Figure 1) [2,4-diamino-5-(3,4,5-trimethoxybenzyl)pyrimidine], is a synthetic antibacterial agent belonging to a group of compounds known as diaminopyrimidines. It was first synthesized as a dihydrofolate reductase inhibitor (DHFR) and used mainly in combination with sulfonamides to treat pneumonia and urinary tract infections. TMP is administered through various routes including intramuscular, intravenous and oral and has high gastro-intestinal tolerability and low side effects. However, low aqueous solubility of TMP [1] reduces the bioavailability from oral formulations. In order to improve TMP dissolution profile, a study conducted by Li *et al.* utilized β-cyclodextrin to form a complex with TMP [2]. The study concluded that the high surface area of contact between TMP and the cyclodextrin along with reduction in drug crystalinity were responsible for improvement in TMP solubility [2]. Nevertheless, the solubility improvement achieved by this study was 2.2 fold. Besides, high doses of β-cyclodextrin are not recommended and can cause diarrhea and bloating as they are degraded and fermented by bacteria in the colon [3]. Therefore, investigation of alternative approaches to improve the solubility of TMP would have a high impact on oral administration of the drug.

Interestingly, TMP has a basic nature (pKa 7.3) and a soluble derivative of the antibiotic can be obtained by reacting TMP with acids resulting in salt formation. Around 50% of the prepared salts of basic drugs are hydrochloride salts. Yet, hydrochloride salts salt out easily and in turn reduce the optimal solubility. Furthermore, hydrochloride salts usually cause high acidity in the formulations and high risk of corrosion [4], while the loss of volatile HCl could affect the stability of weak bases especially over the long-term [5]. Due to the limitations of hydrochloride salts, alternative counter ions that have been explored include citric acid, acetic acid, fumaric acid and maleic acid [5,6,7]. Another class of counter ions that can potentially address the solubility of basic drugs includes acidic amino acids. Fabrizio & Vetere utilized aspartic acid to prepare a water soluble salt of erythromycin. The obtained salt was used to prepare parenteral formulations of erythromycin and was found to be highly tolerable and less toxic [8]. Glutamic acid was used to prepare water soluble salts of chitosan and the glutamate salt was found to improve the apparent permeability of many drugs such as naproxen [9] and rokitamycin [10] when compared to chitosan alone. 

The present study aims to improve the solubility of TMP through salt formation with anionic amino acids, namely glutamic acid and aspartic acid and characterize the prepared salts using FT-IR spectroscopy, proton nuclear magnetic resonance (^1^HNMR), differential scanning calorimetry (DSC) and thermogravimetric analysis (TGA). The study also investigates the effect of different amino acid isomers on the salt characteristics and evaluates the inhibitory effect of the amino acids salts on intrinsically resistant bacteria.

## 2. Materials and methods

### 2.1. Materials

Trimethoprim 98% TLC, L-glutamic acid, L-aspartic acid, D-aspartic acid (99%), D-glutamic acid (minimum 99% TLC) and Potassium bromide (99% FT-IR grade) were purchased from Sigma Aldrich, UK.

D_2_O (99.9% min) was purchased from Coss scientific instrument Ltd. DMSO-D6 (98.8% min) was purchased from MERCK (Darmstadt, Germany). Tryptone Soya Broth (TSB) and Muller Hinton Broth (MHB) were purchased from Oxoid Ltd, Hampshire, England.

### 2.2. Methods

#### 2.2.1. Analytical Technique

The amount of Trimethoprim (TMP) dissolved in the solution samples was quantified using the HPLC (Dionex 1100 system) method reported by Gallego and Arroyo [11]. HPLC was operated at 25 °C on RP-C18, (Phenomenex 110A, 150 × 4.6 mm, 5 µm) column using acetonitrile-NaH_2_PO_4_ buffer (10 mM) (70:30, v/v) (pH 3) as mobile phase which was pumped at 1.0 mL/min flow rate using gradient pump (GP50). UV detector (UVD 170U) was used and the analysis was monitored at 230 nm. The retention time was 1.74 ± 0.037 min and a rectilinear calibration curve was established at concentrations ranging between 10–500 µg/mL (R^2^ of 0.99). LOD and LOQ were calculated using standard deviation of response and slope and were found to be 0.109 and 0.364 respectively.

#### 2.2.2. Phase Solubility Diagram

Phase solubility diagram was established by measuring the saturated solubility of TMP free base with various concentrations of acidic amino acid (L-aspartic acid and L-glutamic acid). Excess TMP was added into screw-capped tubes containing serial dilutions of the amino acid solutions and agitated at room temperature (Stuart SB 162 stirrer) for 24 h. After equilibrium, the supernatant was filtered through 0.45 µm filters and analysed by HPLC to determine TMP concentration. The pH of amino acid solutions was monitored using Xisherbrand Hydrus 500 pH meter.

#### 2.2.3. Salt Preparation

Equimolar amounts of TMP and the free amino acids (L-aspartic acid and L-glutamic acid) were solubilized in water and the solutions were mixed and stirred until equilibration was achieved. The filtrate was transferred into vials and freeze dried for 48 h using a Modulyo freeze dryer at −40 °C shelf temperature and under vacuum. The samples were collected and kept in 40 °C oven for 4h using a Gallenkamp vacuum oven.

Despite the low yield with freeze drying, it is a preferred technique unlike organic solvent methods adapted by Anderson & Conradi [12], as lyophilized products have a high degree of purity and are devoid of the formation of any solvates.

#### 2.2.4. Thermogravimetric Analysis (TGA)

A thermogravimetric analyzer (Pyris 1 TGA, Perkin Elmer) was used in this study to measure the moisture content and decomposition temperature of TMP and its prepared salts. 5–10 mg of samples were loaded on to an open pan and analyzed between temperature range 30–300 °C at 10 °C/min scanning rate and under nitrogen stream. Pyris Manager Software (version 5.00.02) was used to analyze the resultant thermograms.

#### 2.2.5. Differential Scanning Calorimetry

Differential scanning calorimeter (Pyris Diamond DSC) was used to explore thermal events of TMP and its salts. Approximately 2–5 mg of the samples were weighted and transferred to an aluminum sample pan (50 µL capacity). Intra cooler 2P system was used to initially cool the samples to 50 °C and then sample heated to 300 °C at a rate of 10 °C/min. Nitrogen was used as a purge gas at a flow rate of 20 mL/min. Indium and Zinc were used to calibrate the heat flow and melting point onset (156.6 °C for Indium and 419.47 °C for Zinc). The obtained thermograms were analyzed using Pyris Manager Software (version 5.00.02). The experiment was performed in triplicate and an empty aluminum pan was used as a reference cell for all the measurements.

#### 2.2.6. FT-Infrared (IR) Spectroscopy

FTIR absorbance spectra of TMP and its salts were obtained with Nicolet IR 200 spectrometer (Thermo electron corporation) using KBr discs. Crystalline KBr was dried at 65 °C over night before use. Samples were prepared by mixing TMP or its salts with dry KBr at 1:100 (w/w) ratio and pressed at 8 tons for 5 minutes (using Specac tablet presser) to form KBr discs. 64 scans were done over wavelength range of 4000–400 cm^−1^ for each sample. EZ OMNNIC 7.0 software was used to interpret the IR spectrum.

#### 2.2.7. ^1^HNMR

Samples were studied with Bruker Avance DPX-250 NMR (at 250.1 MHz) and Bruker Topspin software was used to analyze the data. Approximately, 1–2 mg of the salts was dissolved in 1 mL of deuterated water (D_2_O) and placed in sample capillary vial. Sample vials were placed into the NMR machine and analyzed for the presence of hydrogen atoms (^1^H-NMR)

#### 2.2.8. pH Solubility Profile

pH solubility studies were determined by using the Ledwidge & Carrigan method [13]. Saturated solution of the salts and acids were prepared by dissolving excess of the drug in deionized water. The solutions were agitated constantly using a stirrer (Stuart SB 162) at ambient temperature. pH was adjusted by adding NaOH or the appropriate acid (L-aspartic acid or L-glutamic acid). After 2 hour interval an aliquot was withdrawn and filtered through 0.45 µm filter, then analyzed using HPLC (see Section 2.2.1)

#### 2.2.9. Solubility and Dissolution Studies

Solubility studies were carried out using Hugchi and Connor’s method [14]. An excess amount of TMP or its salts was added to capped tubes with 5 mL of deionized water and stirred for 24 hours at room temperature until equilibrium was reached. Subsequently, the suspension was filtered through 0.45 μm syringe filters and suitably diluted and the concentration was measured by HPLC.

Hard gelatin capsules were filled with 200 mg of TMP or equivalent amounts of the prepared salts for dissolution studies. USP II paddle method (ERWEKA DT-600) was used to perform the *in-vitro* dissolution studies. The prepared capsules were placed into dissolution vessels containing 900 mL of distilled water (pH 7.2) and the dissolution media was maintained at 37 °C ± 0.5 °C and stirred at 50 rpm. 5 mL of samples were collected at a predetermined time intervals (1, 5, 10, 20, 25, 30, 45 and 60 min) then filtered through 0.45 μm Millipore filters. The dissolution media was replaced by 5 mL of fresh dissolution media in order to maintain a constant volume. After proper dilution samples were analyzed by HPLC as mentioned above.

#### 2.2.10. Microbiological Studies

Two Gram-negative bacterial strains; *Escherichia coli* NCTC 10418 and *Pseudomonas aeruginosa* ATCC 9027 were used for this study. Freshly inoculated bacteria agar plate was prepared one day before the experiment by spreading colony suspension on Trypticase-soy agar plate and incubating for 18 to 24 h.

Zone of inhibition study was performed using Agar plates prepared using Trypticase-soy agar plates. Loopful of bacteria was spread gently on the surface of the agar plates. Six small holes were perforated on the agar plates and filled with 20 µL of 0.5 mM TMP or equivalent concentration of TMP salts. In order to evaluate any inhibitory effect of the amino acids on the bacterial growth, free amino acids were used as controls for this study.

The bacterial minimum inhibitory concentration (MIC) of TMP and its prepared salts against the same two species of gram negative bacteria were performed using micro-dilution method and following the national committee for clinical laboratory standards (NCCLS) specifications.

The bacteria inoculum was established by direct colony suspension method from 18 to 24 h Trypticase-soy agar plates, standardized and counted by Pharmacia spectrophotometer (Pharmacia LKB, Novaspec II). Around 5 × 10^6^ CFU/mL was suspended in Tryptone Soya Broth and used in our study.

50 µL of the bacterial inoculum was transferred to 100 µL of Muller Hinton Broth (MHB) at final concentration ranging between 0.5–0.00024 mM prepared by two fold dilution.

Inoculated plates were incubated at 37 °C for 18–24 h. The lowest concentration of the antibacterial agent that inhibits the growth of bacteria was determined by visual turbidity when compared against control.

#### 2.2.11. Statistical Analysis

Graph Pad Instat® software was used for the statistical analysis study. Data groups were compared using one way analysis of variance (ANOVA) and pair-wise multiple comparisons method (Tukey test). Standard deviation (SD) was used to report the error in the figures and texts. Probability values of 95% (P < 0.05) were used to determine the significant difference.

## 3. Results and Discussion

### 3.1. Phase Solubility Diagram

**Figure 2:**
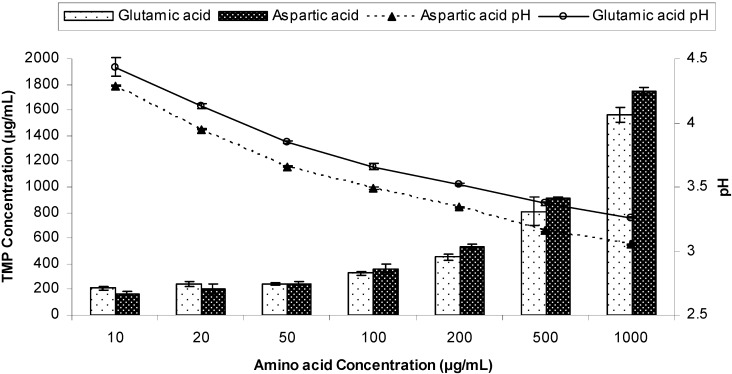
Phase solubility diagram of Trimethoprim (TMP) in the presence of different concentrations of glutamic acid and aspartic acid at different pH (n = 3).

Prior to preparing TMP salts, a solubility phase diagram was first constructed between TMP and two acidic amino acids; aspartic acid and glutamic acid as previously discussed by ElShaer *et al.* [15]. Figure 2 shows that increasing the anionic amino acid concentrations results in lowering of the solution pH and in turn improving the solubility of TMP. Aspartic acid has an acidic (–COOH) side chain with pKa of 1.88 which is 5 units lower than the pKa of TMP (pka 7.3). Therefore TMP could potentially act as a strong base in aspartic acid solution capable of deprotonating the –COOH acidic group. Interestingly, a linear relationship (R^2^ = 0.928) between TMP solubility and aspartic acid concentration was observed which suggests that complete ionization of TMP in aspartic acid solution was achieved suggesting the feasibility of salt formation.

On the other hand, despite the slightly higher pka of the acidic side chain (-COOH) of glutamic acid (pKa = 2.19) a similar trend in solubility improvement was achieved possibly due to the ionization of TMP in solution. These results suggest that both glutamic and aspartic acid can be used in preparing novel salts of TMP.

### 3.2. Characterisation of Salt Form

In order to evaluate the effect of isomer form on the salt characteristics, D and L isomers of glutamic and aspartic acid were used to prepare TMP salts. The prepared salts were further characterized by Fourier transform infra-red, differential scanning calorimetry, thermo-gravimetric analysis and ^1^H nuclear magnetic resonance.

#### 3.2.1. Fourier Transform Infrared Spectroscopy (FT-IR)

FT-IR was performed in order to assess any possible interaction between TMP and the acidic amino acids during salt formation and to investigate any possible changes that could occur due to the use of different isomeric forms of amino acids.

**Figure 3:**
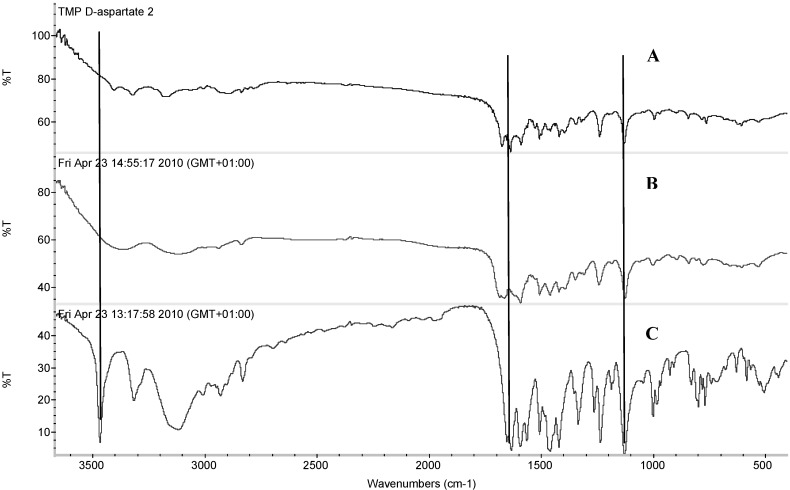
FTIR spectra of (A) trimethoprim D-aspartate, (B) trimethoprim L-aspartate and (C) trimethoprim.

The FT-IR spectra of TMP, TMP L-aspartate and TMP D-aspartate are shown in Figure 3. TMP has characteristic bands at 1634.3 and 1594.7 cm^−1^ which account for deformation of NH_2_ group and stretching of aromation ring respectively. Deformation of C^9^H_2_ group appeared at 1458.7 cm^−1^, while the vibration of C^6^=N of the aromatic primary appeared at 1263.71 cm^−1^. –OCH_3_ aromatic groups had characteristic bands at 1128.68 cm^−1^, as reported by Garnero *et al.* [16].

Assessment of TMP L-aspartate salt showed that TMP bands corresponding to NH_2_ and C-H aromatic stretching vibration were difficult to analyse due to overlapping with NH_2_ and CH aliphatic of the amino acid (Figure 3A). Interestingly, the C=N in the amine aromatic primary became very weak and a new band appeared at 1664.53 cm^−1^ corresponding to νC=O stretching which confirms that the cationic nitrogen has interacted with the carboxylic group of aspartic acid. In contrast, OCH_3_ aromatic groups did not show any changes in the formed salt.

Upon comparing the FT-IR spectra of the L-aspartate and D-aspartate salt, no difference was spotted between the two isomers (Figure 3A & B).

**Figure 4:**
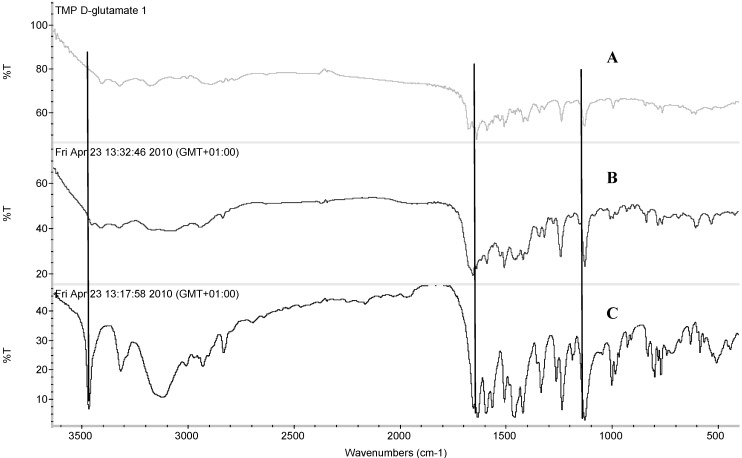
FTIR spectra of (A) trimethoprim D-glutamate, (B) trimethoprim L-glutamate and (C) trimethoprim.

Figure 4 shows the FT-IR for TMP L-glutamate and TMP D-glutamate. Similar to the results obtained for aspartate salts, a new band appeared at 1656.87 cm^-1^ and 1654.74 cm^-1^ for TMP L-glutamate and TMP D-glutamate respectively. This band corresponds to the νC=O of the amino acid carboxylic group which when deprotonated potentially interacts electro-statically with the protonated amino group of TMP prymidine ring. Both the L and D salts had similar FT-IR spectra and no significant difference was observed.

#### 3.2.2. Differential Scanning Calorimetry and Thermogravimetric Analysis

**Figure 5:**
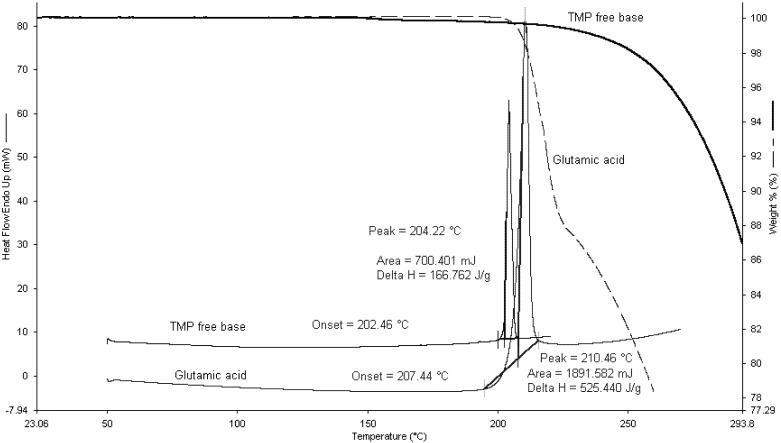
Thermogravimetric analysis (TGA) and Differential Scanning Calorimetry (DSC) scans for L-glutamic acid and TMP free base.

Figure 5 shows the TGA and DSC scans for TMP and L-glutamic acid. TMP had one single endothermic peak at 204.22 °C with an enthalpy of fusion of 166.7 J/g. TGA data revealed that TMP starts to degrade after around 30 °C of its melting.

**Figure 6:**
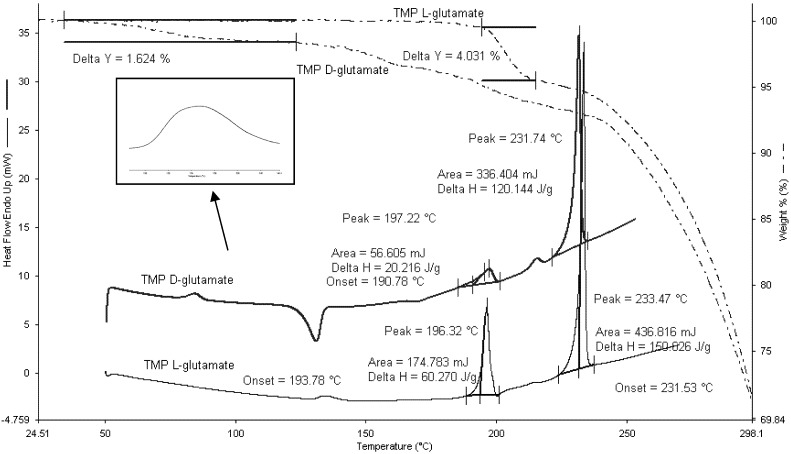
DSC and TGA scans for TMP D-glutamate and TMP L-glutamate salts. 2–5 mg of the sample was heated to 300 °C at rate of 10 °C/min (n = 3).

TMP L-glutamate salt was found to be in the crystalline form as no crystallization exotherm was observed in the DSC scans (Figure 6). At around 130 °C, TMP L-glutamate had an endothermic peak which could possibly correspond to moisture loss. Two endothermic peaks appeared at 197.22 °C (ΔH = 20.22 J/g) and 233.47 °C (ΔH = 150.02 J/g). This data suggests the presence of two enantiotrophs or monotrophs. A second scan was carried out after cooling and the results showed that the first thermal event was irreversible which suggests the presence of monotrophs rather than enantiotrophs. Interestingly, coupling the DSC scans with TGA showed a weight loss (4.03%) at 196 °C which indicates that the thermal event is not a phase transition as polymorphic transitions are not associated with weight loss. In addition, ^1^HNMR data (discussed in details in Section 3.3.3) shows the absence of any impurities which provides further evidence that the weight loss is due to associated water molecules within the crystalline structure of the salt [17].” TGA was carried out to further validate DSC results as mass loss (which is routinely studied with TGA) indicates the presence of moisture [17].

On the other hand, TMP D-glutamate salt is partially crystalline and the amorphous portion of the salt starts to crystallize at 140 °C. Moisture loss appeared as endothermic hump at around 80 °C corresponding to 1.62%. Similar to TMP L-glutamate, TMP D-glutamate showed hydrate loss at 197.22 °C (ΔH = 18.16 J/g) appeared as small peak reflecting the small amount of the hydrate crystals formed during the freeze drying while the rest of the salt was in the amorphous state and crystallizes at 140°C as discussed before. After dehydration, the salt started to melt at 231.74 °C (Figure 6) similar results were suggested by [18].

**Figure 7:**
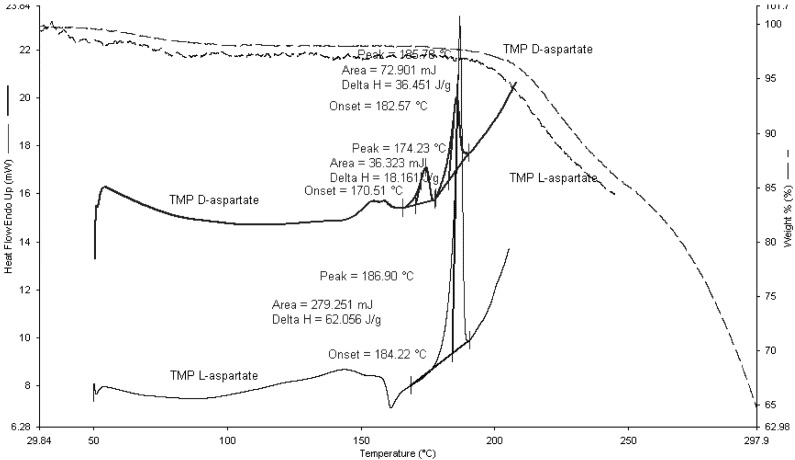
DSC and TGA scans for TMP D-aspartate and TMP L-aspartate. 2–5 mg of the sample was heated to 300 °C at rate of 10 °C/min (n=3).

The thermal events of TMP L-aspartate and TMP D-aspartate were studied using TGA and DSC (Figure 7). TMP L-aspartate showed an up drafted base line between 100–150 °C which corresponds to moisture loss and the salt converted from the amorphous form to the crystalline form at 160 °C followed by crystal melt at 184.22 °C.

#### 3.2.3. ^1^HNMR Studies

The electronic and chemical environment of protons are affected during salt formation which could be reflected by the changes in δ. ^1^HNMR is considered a powerful tool in studying salt formation as it provides insight in to the ratio of molar interaction between the two moieties.

**Figure 8:**
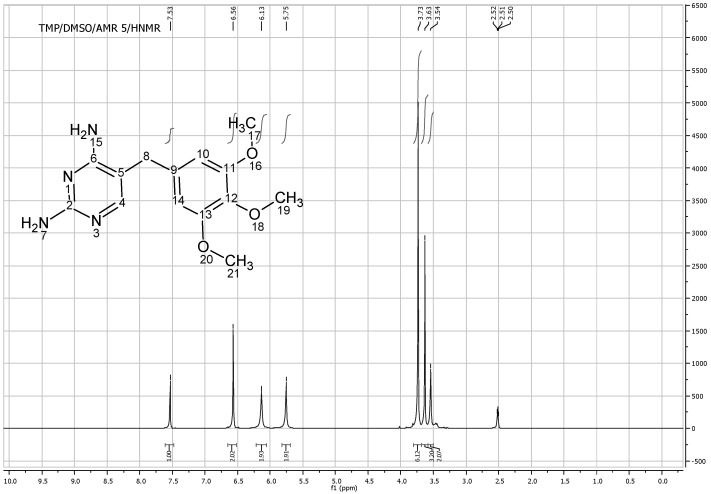
^1^HNMR spectra of trimethoprim free base solubilized in DMSO.

Figure 8 shows the ^1^HNMR of TMP free base (solubilized in DMSO). At 7.53 ppm frequency one proton of TMP heterocyclic ring C^4^H appeared as singlet and the two protons on the aromatic ring C^10^H and C^14^H appeared at 6.56 ppm. The nine hydrogens of OCH_3_ groups appeared as two different peaks; (6H) appeared as a singlet at 3.73 ppm as they have the same surrounding environment, while the other (3H) appeared as a singlet at 3.63 ppm. The two hydrogens of aliphatic (C^8^H_2_) appeared as singlet at 3.54 ppm while two singlet bands appeared at 5.7 and 6.2 which correspond to N^5^H_2 _and N^7^H_2_ respectively. The total integrated H count for TMP was 18 (Figure 8).

**Figure 9:**
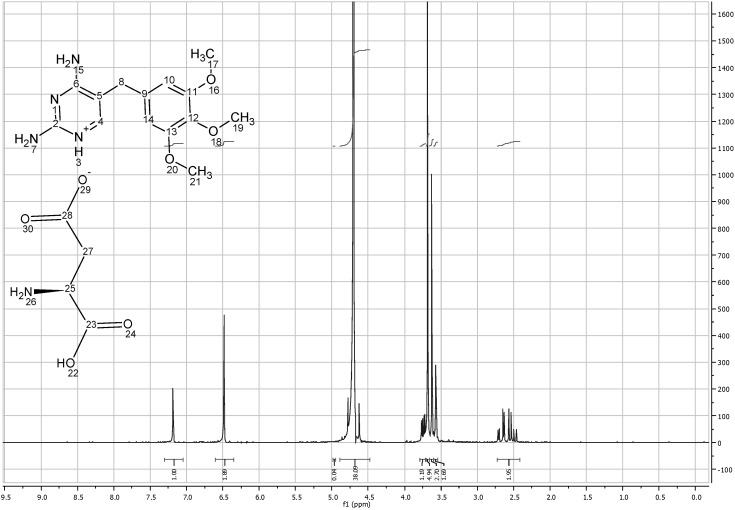
^1^HNMR spectra of trimethoprim L-aspartate solubilized in D_2_O.

TMP L-aspartate salt (solubilized in D_2_O) showed new (3H) assignment at 2.6 corresponding to (C^27^H_2_) and a highly deshielded C^25^H group at 3.75 ppm, which represents the aliphatic chain of L-aspartic acid. As D_2_O was used as a solvent for TMP salts, NH_2_ groups were masked and the total integration of TMP aspartate salt was 17 instead of 21. These results confirm that one mole of L-aspartic acid interacts with 1 mole of TMP to form the salt (*i.e*, 1:1 ratio) as shown in Figure 9.

**Figure 10:**
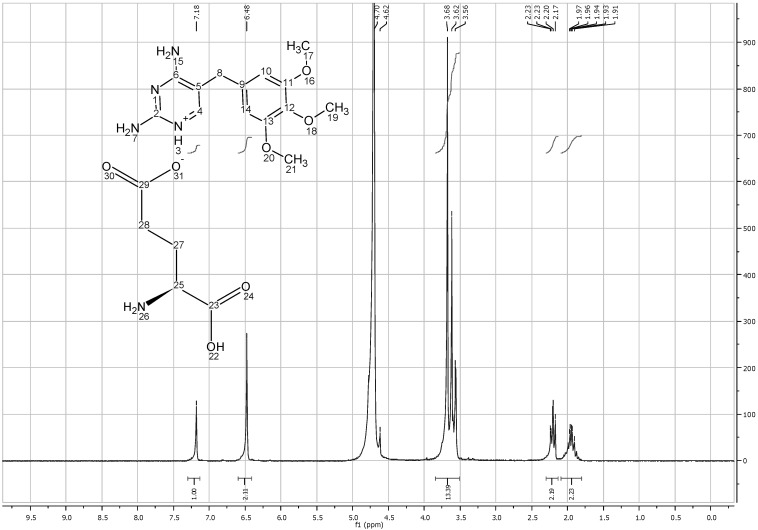
^1^HNMR spectra of trimethoprim L-glutamate solubilized in D_2_O.

^1^HNMR studies of TMP D-aspartate and TMP D-glutamate salts did not reveal any difference in the spectra between the L and D isomers (Figure A & Figure B Appendix 1). The high similarity of the salts formed despite using different isomers suggest that water used to solubilize the amino acids during the preparation step might provide free space for the rotation of the molecules and potentially catalyze racemisation as suggested by [19].

On the other hand, TMP L-glutamate had 5 new protons representing the aliphatic chain of the amino acid. (C^28^H_2_, C^27^H_2_) appeared at 1.96 and 2.2 ppm, while the third C^25^H group appeared at around 3.6 ppm due to the deshielding by NH_2_ group. The total integration also shows that 1 mole of L-glutamic acid interacts with 1 mole of TMP (Figure 10).

#### 3.2.4. Aqueous Solubility and Dissolution Studies

TMP is usually used in combination with sulfonamides in various formulations such as suspension, tablets and solution dosage forms. Therefore TMP solubility and dissolution are important determinants for its bioavailability.

**Figure 11:**
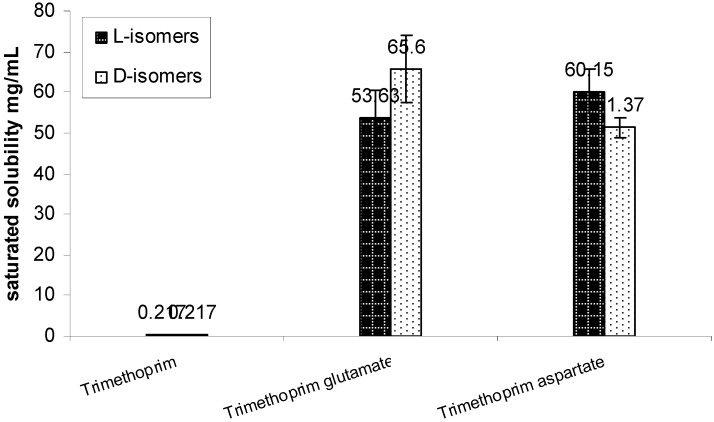
Solubility of trimethoprim and its prepared L & D aspartate and glutamate salts mean ± SD (n = 3).

Solubility studies in our laboratory have found that the saturation solubility of TMP free base was 0.217 mg/mL in water. Using acidic amino acids counter ions improved the solubility of the drug by around 280 fold when compared against the free drug. The solubility of the prepared salts was around 55 mg/mL with no significant difference between the L and D isomers for both the counter ions (Figure 11).

**Figure 12:**
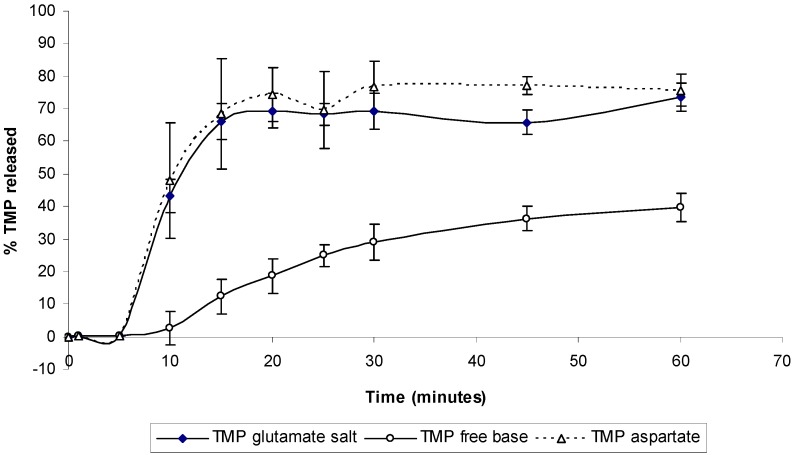
Dissolution profile for TMP free base and its salts in deionized water. Data are mean ± SD (n = 3).

The dissolution profile of TMP and its different salts was also studied (Figure 12). Dissolution studies show a significant improvement of the salt form when compared to the free base. Around 70% of the TMP was released from aspartate and glutamate salt form after only 10 minutes of the dissolution experiment while only 10% release was seen from TMP free form (Figure 12). Such changes in the dissolution profile could be attributed to the ability of TMP salts to exert a self-buffering action, which yield different pH values at the dissolving surface in the diffusion layer [20]. No significant difference was observed between the two salts, which suggest that both salts might have similar buffering capacity and hence similar pH at the diffusion layer.

**Table 1:** pH measurements during solubility and dissolution studies.

		Formulation		
**pH measurement**		TMP free base	TMP Aspartate	TMP glutamate
	Solubility study (Equilibrium)	7.7 ± 0.05	5.075 ± 0.06	5.2 ± 0.11
	End of dissolution study	8.6 ± 0.07	5.37 ± 0.08	5.88 ± 0.05

#### 3.2.5. pH-Solubility Profile

**Figure 13:**
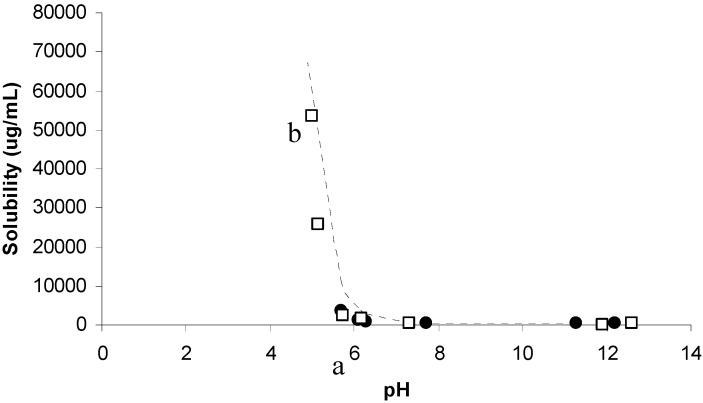
pH solubility profile of TMP and TMP-glutamate salt at ambient conditions using free acid (circles) and TMP-glutamate salt (squares) as starting materials. Points b and a represent the saturation solubility of the salt and TMP free base respectively. [Bs] = 266.35µg/mL and pKa = 7.3.

TMP pka and intrinsic solubility are 7.3 and 0.19 mg/mL respectively. Figure 13 shows the pH solubility profile of TMP when TMP glutamate salt and TMP free base were used as starting materials for determination of solubilities. The intrinsic solubility (Bs) was first determined by increasing the pH of TMP solution to 11.28 using NaOH and Bs was found to be 0.266 mg/mL. When TMP was used as a starting material the solubility was found to increase exponentially upon titrating with glutamic acid (Figure 13). TMP solubility increased from 0.37 mg/mL at pH 7.7 (point a) to 13.8 mg/mL at pH 4.23.

On the other hand, when TMP glutamate salt was used as a starting point, the solubility started to decrease upon titrating with NaOH. TMP glutamate solubility was 53.6 mg/mL at pH 5 and decreased to 0.194 mg/mL at pH 11.9. A similar trend was observed upon titrating TMP with aspartic acid (data not shown). Generally, a good agreement was observed between the experimental values (squares and circles) and the theoretical predicted values (dotted line) see Table (2). Theoretical calculations were carried out using Kramer and Flynn [21] (Equation 1).



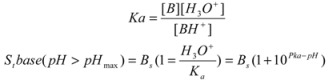
(1)


Where S_t_ is the total solubility at given pH, [B_s_] is the intrinsic solubility of the free base and K_a_ is the acid dissociation constant.

**Table 2:** Theoretical calculations of pH solubility profile using (Equation 1). [Bs] = 266.35µg/mL and pKa =7.3.

pH	Solubility (µg/mL)
11.28	266.35
7.7	372.35
6.3	2929.63
6.1	4487.37
5.7	10869.11
4.9	67165.4

#### 3.2.6. Microbiology Studies

After having significantly improved both the solubility as well as the dissolution characteristics using the salt forms of TMP, the next stage of the work was to evaluate microbiological properties to study if the therapeutic effect of the drug was still retained upon preparation of the different salts.

Interestingly, zone of inhibition studies showed that use of amino acid salts not only retained the anti-bacterial activity against TMP sensitive species; *Escherichia coli* but also had a significant increase in the total area of inhibition (Table 3). To rule out the possible effects of amino acids alone on bacterial growth inhibition, control experiments with the amino acids were performed which resulted in no inhibition of bacterial growth on the plates. Therefore, the higher zone of inhibition can be attributed to the significant improvement in solubility which potentially results in higher permeability across the agar plates resulting in larger zone of inhibition. This could possibly translate into a faster therapeutic effect along with reduced therapeutic dose in a clinical setting.

On the other hand, neither the drug nor the salts showed any activity against TMP resistance species *Pseudomonas aeruginosa.* This is probably due to the complex structure of *Pseudomonas aeruginosa* membrane as it is intrinsically resistant to TMP and prevents the entry of both the drug and amino acid salts.

**Table 3:** Zone of inhibition studies of TMP and its prepared salts against *Escherichia coli* and* Pseudomonas aeruginosa.*

	*Escherichia coli*		*Pseudomonas aeruginosa*
	*Area* (cm^2^)	*% compare to the total plate area*	
TMP	4.03 ± 0.2	18.7	****
TMP aspartate	4.6 ± 0.2	21.4	****
TMP glutamate	4.65 ± 0.2	21.6	****

Bacterial minimum inhibitory concentration was carried out as well in order to accurately determine the MIC of the TMP and its salts. Two species were used in this study; *Escherichia coli* and the intrinsically resistant organism, *Pseudomonas aeruginosa*. The MIC of TMP on *E. coli* was 0.00048 mM and TMP aspartate and glutamate salt had similar MIC values which confirm our previous finding that salt preparation did not impair the pharmacological function of TMP (Table 4).

**Table 4:** MICs of TMP and its prepared salts against *Escherichia coli and Pseudomonas aeruginosa.*

	*Escherichia coli*	*Pseudomonas aeruginosa*
TMP	0.00048 mM.	>0.5 mM
TMP aspartate	0.00048 mM	>0.5 mM
*TMP glutamate*	0.00048 mM	>0.5 mM

## 4. Conclusion

TMP is a basic synthetic antibacterial agent which is poorly water soluble [1,2]. In order to improve its solubility, the ability of TMP to form new salts with L and D isomers of acidic amino acids was investigated in this study. The study has demonstrated that TMP solubility increased significantly upon coupling with aspartic and glutamic acid and this increase was attributed to the novel salt formation which has self-buffering capacity yielding favorable pH for salt solubility during dissolution. Moreover, the antibacterial activity of these new salts was investigated and no change was seen in their MIC when compared against TMP free base. Using different isomers; l- and d- forms did not show any significant difference in the salt characteristics. Our future work will investigate the effect of amino acids salts on the absorption characteristics of TMP across intestinal membrane using Caco-2 cells.

## Figures and Tables

**Figure A:**
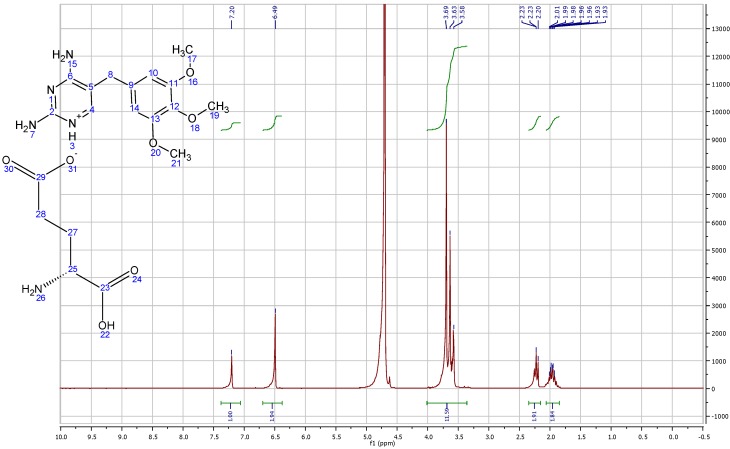
^1^HNMR spectra of trimethoprim D-glutamate solubilized in D2O.

**Figure B:**
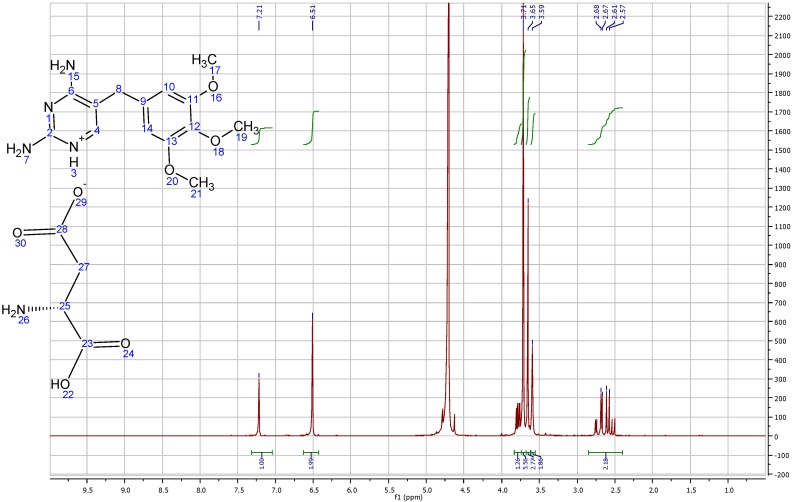
^1^HNMR spectra of trimethoprim D-aspartate solubilized in D2O.
